# Selection of Non-Mycotoxigenic Inulinase Producers in the Group of Black Aspergilli for Use in Food Processing

**DOI:** 10.17113/ftb.60.04.22.7521

**Published:** 2022-12

**Authors:** Sanja Stojanović, Jelena Stepanović, Bojana Špirović Trifunović, Nataša Duduk, Biljana Dojnov, Bojan Duduk, Zoran Vujčić

**Affiliations:** 1 University of Belgrade, Institute of Chemistry, Technology and Metallurgy, National Institute of the Republic of Serbia, Department of Chemistry, Njegoševa 12, 1100 Belgrade, Republic of Serbia; 2 Institute of Pesticides and Environmental Protection, Banatska 31/b, 11080 Belgrade, Republic of Serbia; 3 University of Belgrade, Faculty of Agriculture, Nemanjina 6, 11080 Belgrade, Republic of Serbia; 4 University of Belgrade, Faculty of Chemistry, Department of Biochemistry, Studentski trg 12-16, 11000 Belgrade, Republic of Serbia

**Keywords:** ochratoxin, fumonisin, *Aspergillus* spp., fructooligosaccharides, inulinase

## Abstract

**Research background:**

Inulinases are used for fructooligosaccharide production and they are of interest for both scientific community and industry. Black aspergilli represent a diverse group of species that has use for enzyme production, in particular some species are known as potent inulinase producers. Finding new potential producers from the environment is as important as improving the production with known strains. Safe use of enzymes produced by aspergilli in food industry is placed ahead of their benefit for inulinase production.

**Experimental approach:**

Here we show a specific approach to finding/screening of newly isolated fungal inulinase producers that combines a newly developed screening method and an equally important assessment of the toxigenic potential of the fungus. In this study 39 black aspergilli collected from different substrates in Serbia were identified and assessed for inulinase production.

**Results and conclusions:**

The most common species were *Aspergillus tubingensis* (51.2%), followed by *A. niger* (23.1%), *A. welwitschiae* (23.1%) and *A. uvarum* (2.6%). The isolates for inulinase production were selected using a cheap and easy, fast and non-hazardous alternative inulinase screening test developed in this work. Enzymatic activity of selected inulinase-producing strains was confirmed spectrophotometrically. Since some *A. niger* and *A. welwitschiae* strains are able to produce mycotoxins ochratoxin A (OTA) and fumonisins (FB), the toxigenic potential of selected inulinase producers was assessed analytically and genetically. Fungal enzyme producer can be considered safe for use in food industry only after comparing the results of both approaches for investigating toxic potential, the direct presence of mycotoxins in the enzyme preparation (analytically) and the presence of mycotoxin gene clusters (genetically). In some strains the absence of OTA and FB production capability was molecularly confirmed by the absence of complete or critical parts of biosynthetic gene clusters, respectively. The two best inulinase producers and mycotoxin non-producers (without mycotoxin production capability as additional safety) were selected as potential candidates for further development of enzyme production.

**Novelty and scientific contribution:**

The presented innovative approach for the selection of potential fungal enzyme producer shows that only non-toxigenic fungi could be considered as useful in food industry. Although this study was done on local isolates, the approach is applicable globally.

## INTRODUCTION

*Aspergillus* section *Nigri* (the black aspergilli) consists of a number of species rich in variety and number of enzymes and secondary metabolites, frequently used in biotechnological processes and food technology. On the other hand, many species can cause food spoilage on a wide variety of food substrates (soil, nuts, fruits, vegetables, meat and dairy products), and even human diseases (usually of immunocompromised patients). Moreover, they are typical, commonly present indoor fungi (*1*, *2*). The most commonly occurring and also the most important industrial species is *Aspergillus niger*. It has a long history of use for enzyme production (*3*). Some black aspergilli are known as potent inulinase producers (*4*, *5*). Inulinases are used for production of valuable prebiotics – fructooligosaccharides (FOS). A prebiotic is defined as a ’nondigestible compound that, through its metabolization by microorganisms in the gut, modulates the composition and/or activity of the gut microbiota, thus conferring a beneficial physiologic effect on the host’ (*6*). Applications of FOS are widespread: food and beverages (dairy products, baked goods, fermented meat, dry foods), as dietary supplements (food, nutrition, infant formulation) and in animal feed. FOS are used especially in combination with artificial high-intensity sweeteners, improving their sweetness and aftertaste. In human studies the tested daily doses of FOS vary in the range of 5–20 g, which represents 1–4% of the total food intake (estimations based on World Health Organization recommended daily food intake) (*7*). Prevalence of prebiotics in human diet is insufficient worldwide and therefore there is a need to increase their production and application in food products. The increase in the functional food market, specifically with regards to foods that contain prebiotics, has been tremendous over the last two decades. Globally, the prebiotic market is expected to continue to grow in the next years (*8*). This implies the need for research of new and improved inulinases. The pursuit of novel enzymes has prompted researchers to explore native fungal isolates from different habitats, which is often an important starting point (*9*, *10*).

The screening of potential producers among fungi starts with the isolation of fungi from different habitats, cultivation on substrates for inducible enzymes and detection of enzymatic activity in the obtained fermentation extracts, and therefore studies usually analyze a small number of fungal isolates (*9*, *10*). Another approach is based on direct selection during fungal growth on substrates for inducible enzymes (*in situ*) where results are evaluated in terms of colony growth by visual analysis of the formed mycelium (*11*) or by dyeing of nonhydrolyzed inulin after fungal growth (*12*). Both approaches for inulinase producer selection imply a long fungal growth period. It would be helpful to find suitable methods for selection among a high number of species. Fungi are to be grown for as short period as possible to avoid sporulation, which occurs in *Aspergillus* sometimes within just 48 hours. Enzyme activity is to be detected in a high number of extracts after fermentation as simply as possible and with great accuracy.

Black aspergilli share morphological features which makes their classification and identification difficult and therefore the taxonomy of the section *Nigri* has been revised several times (*13*). Currently, identification of *Aspergillus* species is based on a polyphasic approach which includes molecular, morphological and extrolite analyses (*13*, *14*). Morphologically similar or sometimes undistinguishable species, grouped into *Aspergillus niger* ’aggregate’, can only be reliably identified based on molecular features, *i.e.* by analysis of the calmodulin (*CaM*) gene (*14*).

Apart from the favourable, direct economic effect of black aspergilli, some of them are mycotoxin-producing species. Mycotoxins are low-molecular-mass secondary metabolites produced by filamentous fungi which are acutely or chronically toxic and pose a health hazard to humans and vertebrates when acquired in small amounts orally, by inhalation, or *via* the skin (*15*). They are important for food safety and also for biotechnological processes since they represent a safety concern in the industrial application of fungi for enzyme or bulk metabolite production. Only non-toxicogenic strains can be used as source organisms in the production of enzyme preparations for use in food (*16*). For a long time, *A. niger* has been considered non-toxic in industrial conditions and is therefore Generally Recognized as Safe (GRAS) (*17*). While that remains true for some of the industrial strains, for others toxigenicity has been revealed and wild-type *A. niger* is considered by German authorities (BAUA, *i.e.* German Federal Institute for Occupational Safety and Health) as a class 2 biological agent because of its potential to produce mycotoxins and cause disease in humans and animals (*15*, *18*). The main mycotoxins produced by *Aspergillus niger* and its closely related species *A. welwitschiae* are ochratoxin A (OTA) and fumonisins (FB) (*15*, *18*, *19*). OTA and FB are classified by the International Agency for Research on Cancer (IARC) as a possible human carcinogen (group 2B) (*20*). For a long time, *Fusarium* species had been known as producers of fumonisins (*21*), while for *A. niger* sequencing of the full genome in 2006 and discovery of a putative *fum* gene cluster indicated its potential to produce fumonisins (*22*, *23*). Later, production of fumonisins by some industrial strains of *A. niger* and by some isolates from coffee beans has been reported (*24*–*26*), as well as by some *A. welwitschiae* strains (*27*). Not all isolates of these species produce mycotoxins, and their frequency in population can vary. The toxigenic potential is genetically encoded by the fumonisin and ochratoxin biosynthetic gene clusters (*28*, *29*).

There are limited data on the diversity of black aspergilli in Serbia, while no information on their OTA and FB toxigenicity and the associated risk are known. This information is particularly important having in mind that in recent studies in Serbia the most prevalent mycotoxins in plant-based animal feed were OTA and fumonisins, present in 91.4 and 68.6% of tested samples, respectively (*30*). It is not enough to determine whether a fungal enzyme producer produces hazardous mycotoxins under specified growth conditions; it is also important to examine whether there is a genetic potential for their production. Therefore, enzyme producers can be either selected based on the absence of genes involved in biosynthesis of mycotoxins or developed by deletion of the genes through mutagenesis (*15*, *18*).

Screening for potential fungal inulinase producers and their safe utilization, particularly for food preparation (FOS), should only consider enzyme producers that are nontoxigenic. Therefore, the aims of this study are to: (*i*) identify black aspergilli isolated from different substrates in Serbia, (*ii*) develop a fast and unambiguous screening test to assess their inulinase production, and (*iii*) evaluate their mycotoxin-producing potential genetically and using analytical techniques.

## MATERIALS AND METHODS

### Isolation and identification of fungal strains

All reagents and solvents used in this study were of the highest available purity and were purchased from Sigma-Aldrich, Merck KGaA (Darmstadt, Germany) Qiagen (Hilden, Germany) and Biolife (Milano, Italy). Black mould was isolated from different substrates (Table 1) from 2000 to 2016 using standard microbiological techniques by transferring conidia on potato dextrose agar (PDA) in Petri dishes and incubating at 28 °C for 7 days. Following incubation, the plates were examined, and fungal spores were transferred onto new PDA. Pure cultures were preserved in glycerol stocks and stored in laboratory strain bank UB483 at −70 °C.

**Table 1:** *Aspergillus* section *Nigri* isolates used in this study and their inulinase activities

Isolate	Geographic origin	Source	Year of isolation	Identification	Inulinase screening test	Inulinase activity/(U/mL)*
FAT 1	Belgrade	malt agar	2000	*A. tubingensis*	-	n.t.
FAT 2	Belgrade	orange	2000	*A. tubingensis*	-	n.t.
FAT 25	Grabovac	bread	2001	*A. tubingensis*	-	n.t.
FAT 26	Belgrade	bread	2001	*A. tubingensis*	-	n.t.
FAT 27	Belgrade	bread	2001	*A. tubingensis*	+	0.098±0.002
FAT 28	Grabovac	bread	2001	*A. tubingensis*	-	n.t.
FAT 29	Herceg Novi, Montenegro	bread	2001	*A. tubingensis*	+	0.101±0.003
FAT 30	Belgrade	bread	2001	*A. tubingensis*	+	0.067±0.006
FAT 31	Belgrade	bread	2001	*A. tubingensis*	+	0.123±0.003
FAT 32	Belgrade	bread	2001	*A. tubingensis*	+	0.119±0.002
FAT 33	Belgrade	bread	2001	*A. tubingensis*	-	n.t.
FAT 34	Belgrade	bread	2001	*A. tubingensis*	+	0.075±0.003
FAT 35	Belgrade	bread	2002	*A. tubingensis*	+	0.104±0.002
FAT 36	Grabovac	marmalade	2002	*A. tubingensis*	-	n.t.
FAT 37	Petrovac, Montenegro	bread	2002	*A. tubingensis*	-	n.t.
FAT 38	Belgrade	bread	2008	*A. tubingensis*	+	0.096±0.004
FAT 39	Belgrade	cellulose	2016	*A. tubingensis*	+	0.122±0.002
FAT 40	Belgrade	chestnut	2016	*A. tubingensis*	-	n.t.
FAT 41	Belgrade	rice	2016	*A. tubingensis*	+	0.193±0.005
FAT 42	Belgrade	cereals mix	2009	*A. tubingensis*	-	n.t.
FAN 1	Belgrade	lemon	2011	*A. niger*	+	0.079±0.007
FAN 2	Belgrade	bread	2002	*A. niger*	-	n.t.
FAN 3	Belgrade	bread	2002	*A. niger*	+	0.064±0.011
FAN 4	Prague, Czech Republic	bread	2011	*A. niger*	-	n.t.
FAN 5	Belgrade	Starch and soya extract mixture	2010	*A. niger*	+	0.118±0.007
FAN 6	Belgrade	*Sambucus racemosa* extract	2012	*A. niger*	-	n.t.
FAN 8	Belgrade	corn flour	2016	*A. niger*	+	0.180±0.009
FAN 9	Belgrade	corn flour	2016	*A. niger*	+	0.140±0.007
FAN 10	Belgrade	rice	2016	*A. niger*	+	0.163±0.005
FAW 1	Zemun	bread	2001	*A. welwitschiae*	+	0.472±0.004
FAW 2	Belgrade	bread	2001	*A. welwitschiae*	+	0.127±0.005
FAW 3	New Belgrade	clindamycin capsule	2001	*A. welwitschiae*	+	0.144±0.007
FAW 4	Belgrade	lemon	2001	*A. welwitschiae*	+	0.135±0.006
FAW 5	Krajišnik	plum marmalade	2002	*A. welwitschiae*	-	n.t.
FAW 6	Belgrade	wheat straw	2009	*A. welwitschiae*	+	0.468±0.002
FAW 7	Belgrade	artichoke silage	2012	*A. welwitschiae*	-	n.t.
FAW 8	Belgrade	gelatin	2016	*A. welwitschiae*	-	n.t.
FAW 9	Belgrade	tomato juice	2002	*A. welwitschiae*	+	0.366±0.004
FAU 1	Belgrade	soil	2012	*A. uvarum*	-	n.t.

*Each data point represents the mean of two independent assays±SEM (standard error of the mean). n.t.=not tested

**Fig. 1:**
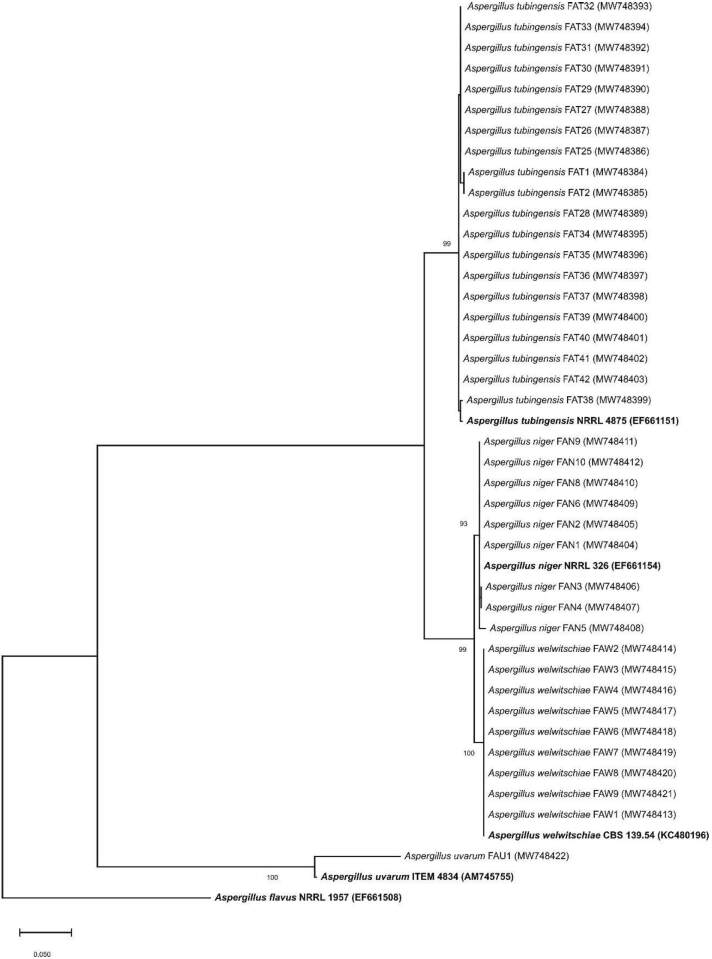
The evolutionary history was inferred by using the Maximum Likelihood method and Kimura 2-parameter model (*36*) of 39 sequences of black aspergilli obtained in this work, reference strains, and *Aspergillus flavus* as the outgroup. The tree with the highest log likelihood is shown. Strain names and accession numbers are given in parentheses. The reference strains are in bold. The tree is drawn to scale, with branch lengths measured in the number of substitutions per site

DNA was extracted from the isolates grown on PDA for 2 days at 28 °C. Genomic DNA was extracted from white mycelium using the cetyltrimethylammonium bromide (CTAB) protocol of Day and Shatttock (*31*). Total DNA was precipitated with isopropanol, resuspended in TE buffer and stored at -20 °C until use. For identification, DNA was amplified with the primer pair CMD5/CMD6, based on *CaM* gene (*32*). PCR reaction mix (25 μL) contained 1 µL of template DNA, 1×PCR MasterMix (Thermo Fisher, Vilnius, Lithuania) and 0.4 μmol/L of each primer. The amplification parameters were as described previously (*32*). Samples without DNA were used as negative control. PCR products were observed on a 1% agarose gel, stained in ethidium bromide and visualized with UV transilluminator. Amplified CMD5/CMD6 product of each isolate was directly sequenced in both directions using the same primers as for the amplification. The obtained sequences were assembled using Pregap4 from the Staden program package (*33*), and compared with sequences publicly available using the MegaBlast algorithm (*34*). They were aligned with those of the strains representing the closest species using ClustalX (*35*), under MEGA X (*36*). Evolutionary history was inferred based on the partial *CaM* gene sequences of black aspergilli obtained in this study, reference strains and *Aspergillus flavus* as outgroup, using the Maximum Likelihood (ML) method (MEGA X). A sequence evolution model was first chosen using the “find best model” option in MEGA X. Initial tree(s) for the heuristic search were obtained automatically by applying Neighbor-Join and BioNJ algorithms to a matrix of pairwise distances estimated using the Maximum Composite Likelihood approach, and then selecting the topology with superior log likelihood value. To estimate the statistical significance of the inferred clades, 1000 bootstraps were performed (Fig. 1).

### Screening of fungal inulinase producers

Fungal isolates were grown in solid medium (modified Czapek solution without sucrose, Jerusalem artichoke extract 167 g/L and agar-agar 15 g/L) for 33 h at 28 °C. Jerusalem artichoke was purchased from the local market (Belgrade, Serbia). Jerusalem artichoke exudate was prepared by homogenization of artichoke tubers in a blender to obtain all the liquid (rich in inulin) and the dry residues were discarded. Fermentation extracts for enzyme analysis were obtained by extraction with 25 mmol/L acetic buffer, pH=5.0, and shaking for 2 h at 150 rpm, with subsequent centrifugation and microfiltration.

**Fig. 2:**
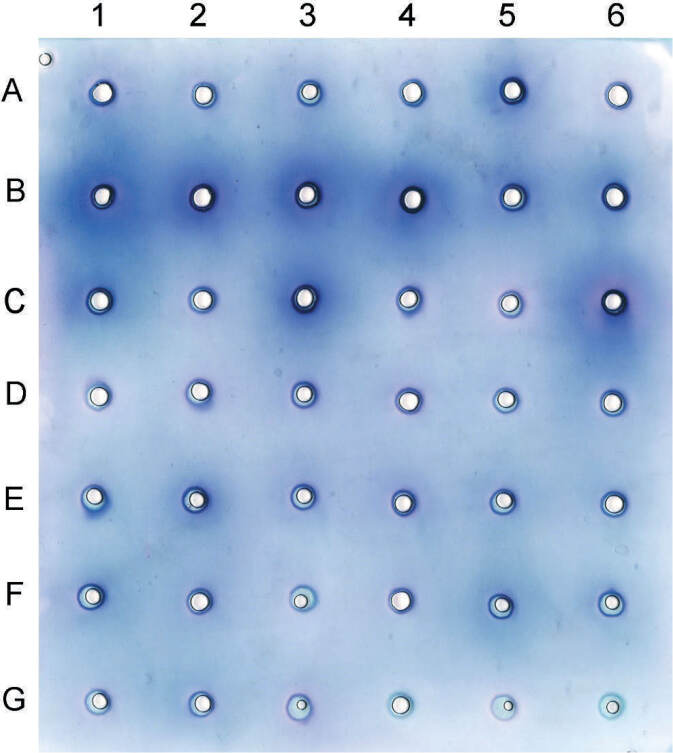
Screening test for potential inulinase producers among *Aspergillus* isolates on polyacrylamide gel with incorporated inulin. Hydrolyzing products were detected by NBT reagent. Isolates were identified in the following order: from A1 to B3 as *A. niger* (FAN1, 2, 3, 4, 5, 6, 8, 9 and 10), from B4 to C6 as *A. welwitschiae* (FAW1, 2, 3, 4, 5, 6, 7, 8 and 9), D1 as *A. uvarum* (FAU1), from D2 to G3 as *A. tubingensis* (FAT1, 2, 25, 26, 27, 28, 29, 30, 31, 32, 33, 34, 35, 36, 37, 38, 39, 40, 41 and 42), and from G4 to G6 are denatured enzymes used as negative control

Inulinase diffusion was screened in a 6% polyacrylamide (PAA) gel containing an incorporated substrate (20 g/L inulin). Samples of 20 μL (fermentation extracts) were added to the wells in the gel with an interval of 5 s and the reaction was carried out in a humid environment at 37 °C for 18 h. The gel was stained by nitroblue tetrazolium (NBT) reagent in 0.3 mol/L NaOH. The resulting violet colouring around the wells indicates the existence of inulin hydrolysis products – reducing sugars (Fig. 2).

Inulinase activity was assayed according to the dinitrosalicylic acid (DNS) procedure (*37*). Inulin (5 g/L) was used as substrate, and the reaction was performed at pH=4.5 and 37 °C for 2 h. Absorbance of reaction products was measured spectrophotometrically at 540 nm (UV-1800 UV/Vis spectrophotometer; Shimadzu, Kyoto, Japan). All enzyme assays were done in duplicate and represented as mean value±SEM (standard error of the mean). One unit of enzyme activity, U/mL, was defined as the amount of enzyme required to produce 1 μmol of the reaction product in 1 min under the conditions given above.

### Mycotoxin gene cluster analyses

**Fig. 3:**
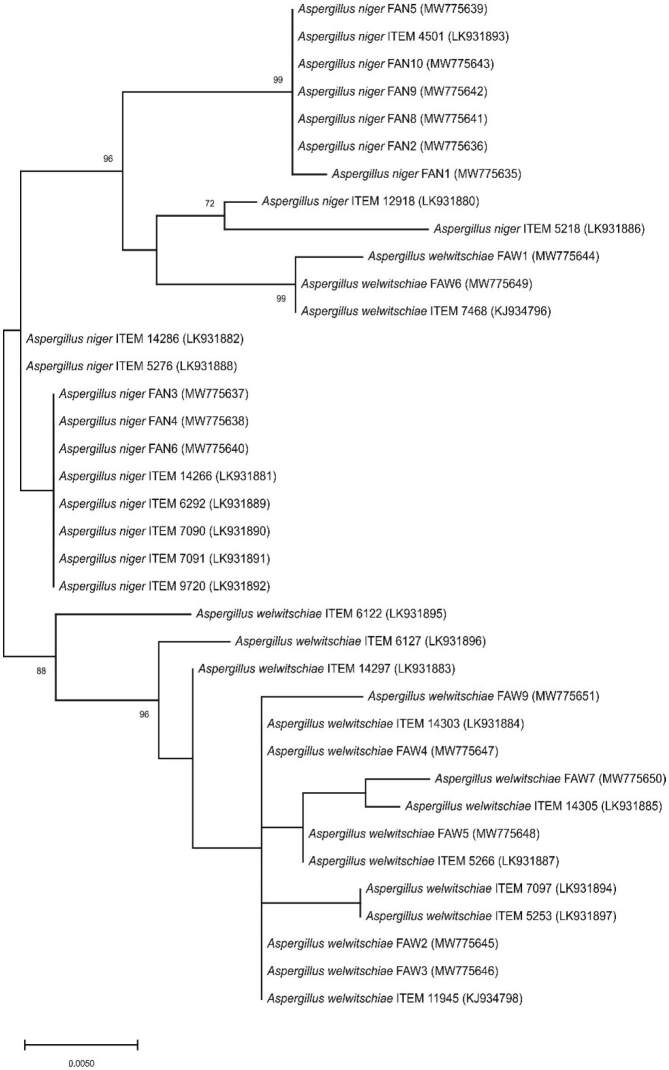
The evolutionary history was inferred by using the Maximum Likelihood method and Kimura 2-parameter model (*36*) of 17 *fum1* sequences of black aspergilli obtained in this work. The tree with the highest log likelihood is shown. Strain names and accession numbers are given in parentheses. The tree is drawn to scale, with branch lengths measured in the number of substitutions per site

Fumonisin cluster genes *fum1, fum7, fum10, fum15* and *fum21* (2 regions designated I and II) were amplified using primer pairs and the amplification parameters as described by Susca *et al.* (*28*). Ochratoxin cluster genes *ota1, ota2, ota3* and *ota5* were amplified using primer pairs and the amplification parameters as described by Susca *et al.* (*29*). A confirmed ochratoxin producer (data not shown), an *A. welwitschiae* isolate from the collection of the Postharvest laboratory (University of Belgrade, Faculty of Agriculture), was used as positive control in the PCRs for amplification of *ota* genes. PCR reaction mix, negative controls and PCR product observation were as described for amplification of *CaM*. The presence of amplicons of appropriate size was considered as positive reaction. Amplification products of *fum1* of all analyzed strains were sequenced in both directions with the primers applied for amplification (Macrogen Inc., Seoul, Korea). The obtained *fum1* sequences were assembled, manually inspected, compared as described for *CaM*, and were deposited in the NCBI GenBank (*38*) under accession numbers given in Fig. 3. The sequences were aligned with those publicly available from the previously described strains using Clustal X, under MEGA X (*29*, *35*, *36*). Evolutionary history was inferred based on the *fum1* sequences of strains (*A. niger* and *A. welwitschiae*) from this study and 20 previously published and toxigenically characterized *A. welwitschiae* and *A. niger* strains, using the ML method, as described for *CaM*.

### Mycotoxin analyses

#### Determination of mycotoxin and inulinase production

All *A. welwitschiae* isolates were grown for 96 h on coarse ground triticale grains, at 28 °C and relative humidity (RH) 60% in solid-state fermentation (SSF) in a Thermostat chamber (HPP IPP plus; Memmert USA, LLC; Eagle, WI, USA). Triticale (×*Triticosecale* sp.) “Odisej” line was obtained from the Institute of Field and Vegetable Crops, Novi Sad, Serbia. After 96 h, the substrates were covered with white mycelium and there was no sporulation. Fermentation extracts for mycotoxin analysis were obtained by extraction with 100 mL of 25 mmol/L acetic buffer, pH=4.5, and shaking for 2 h at 150 rpm, with subsequent centrifugation at 13 257x*g* (Thermo Scientific SL40R; Thermo Fisher Scientific, Waltham, MA, USA) and microfiltration (glass microfiber filters GF/B, 1.0 μm; Whatman, GE Healthcare Life Sciences, Buckinghamshire, UK). Protein concentrations in fermentation extracts were determined according to Bradford (*39*). Inulinase activity was assayed as described above (section *Screening of fungal inulinase producers*), at 50 °C for 15 min. Specific inulinase activity was defined as U per mg of proteins. Productivity of inulinase during fermentation was defined as U/(mL·h).

#### LC-MS/MS analysis of mycotoxins

All reagents were of HPLC grade and purchased from Sigma-Aldrich, Merck KGaA. OTA, FB1 and FB2 were extracted by modified QuEChERS (Quick, Easy, Cheap, Effective, Rugged and Safe) procedure (*40*). After centrifugation at 4816x*g* (Thermo Scientific SL40R; Thermo Fisher Scientific) for 5 min, 1 mL of acetonitrile supernatant was filtered through a 0.45-µm syringe filter prior to LC-MS/MS analysis of OTA. For FB1 and FB2 determination, 1 mL of supernatant was evaporated in a stream of nitrogen and then reconstituted in 1 mL of *φ*(acetonitrile,water)=50%. LC-MS/MS conditions were optimized using MassHunter Optimizer software (*41*), based on quantitation/qualification ion 239/221 for OTA, which resulted in fragmentation and collision energy of 105/120 and 20/33 V, respectively. Linearity of detector response was investigated based on solvent (acetonitrile) and matrix match calibrations with U-[13C20]-OTA as internal standard. Quantitation/qualification ion 353.3/334.4 for FB1 resulted in fragmentation and collision energy of 140/140 and 40/40 V, and ion 336.3/318 for FB2 in fragmentation and collision energy of 140/140 and 35/35 V, respectively. Linearity of detector response was investigated based on solvent (acetonitrile/water) and matrix match calibrations with U-[13C34]-FB1 as internal standard. For LC analysis, an Agilent 1260 Infinity (Agilent Technologies, Santa Clara, CA, USA) HPLC system with a binary pump was used, equipped with a reversed-phase C18 analytical column of 50 mm×4.6 mm and 1.8 µm particle size (Zorbax Eclipse XDB-C18; Agilent Technologies). The mobile phase was Milli-Q water (solvent A) and methanol (solvent B), both containing 0.02 mol/L formic acid in gradient mode (5% at 0 min, 50% for 5 min, 98% for 20 min), with a flow rate of 0.3 mL/min. The injection volume was 10 μL. For mass spectrometry analysis, an Agilent 6460 Triple-Quad LC/MS system was applied and Agilent MassHunter B.06.00 software (*41*) was used for data acquisition and processing. Analysis was performed in the positive ion mode for both OTA and fumonisins. The electrospray ionization (ESI) source values were as follows: drying gas (nitrogen) temperature 300 °C, drying gas flow rate 5 L/min, nebulizer pressure 310.3 kPa and capillary voltage 3500 V. Mycotoxins were detected using the dynamic multiple reaction monitoring mode (dMRM).

### Statistical analysis

All inulinase activity tests in *Screening of fungal inulinase producers* were performed in duplicate and the results are presented as mean value±SEM (standard error of the mean). All inulinase activity tests in Mycotoxins and inulinase production section were performed in triplicate and the results are presented as mean value±S.D. (standard deviation). One-way ANOVA with *post hoc* multiple comparison tests (Tukey’s test; p<0.05) were used to define statistical differences among produced enzymes using GraphPad Prism v. 8.4.3. software (*42*).

## RESULTS AND DISCUSSION

### Identification of fungal strains

A total of 39 pure culture isolates from different substrates (Table 1) showing morphological features of black aspergilli (fast growing with abundant dark brown to black sporulation) were subjected to molecular identification. Using the CAM5/CAM6 primer pair in the PCR, the expected length amplicons (~0.6 kb) were obtained with all studied isolates, while no amplification was obtained for negative control (data not shown). Direct sequencing of the obtained calmodulin amplicons yielded nucleotide sequences (528-560 nt long), which were deposited in the NCBI GenBank (*38*) under accession numbers given in Fig. 1. In the phylogenetic analyses of the obtained *CaM* amplicon sequences and the representative strains of the closest species, Serbian black aspergilli isolates clustered within the *Aspergillus nigri* clade and they formed four highly supported separate subclusters with representatives of four species, thereby confirming identification of 20 isolates as *A. tubingensis*, nine as *A. welwitschiae*, nine as *A. niger*, and one as *A. uvarum* (Fig. 1).

Black aspergilli represent a diverse group of species commonly present on different substrates (*^(^**^1^**^)^*). In this study, isolates of black aspergilli collected for several years and from different substrates in Serbia were identified as belonging to four *Aspergillus* species, based on the morphology and *CaM* phylogeny proposed by Samson *et al.* (*14*). The most common was *A. tubingensis* (more than half) followed by *A. niger* and equally frequent *A. welwitschiae*, and the least frequent was *A. uvarum*.

### Inulinase producer screening

All fungal isolates were grown on modified Czapek’s agar, in which inulin extracted from artichoke tubers replaced sucrose, for 33 h without signs of sporulation. A quality diffusion test for inulinase activity was developed. The quantity of added enzyme, substrate concentration and reaction time were optimized. A total of 39 isolates from different substrates (shown in Table 1) were tested. Results of enzyme screening demonstrated that 22 isolates were positive in the diffusion test: ten *A. tubingensis* isolates, six *A. niger* isolates and six *A. welwitschiae* isolates (Fig. 2).

Enzymatic activity was quantified spectrophotometrically (UV-1800 UV/Vis spectrophotometer; Shimadzu) only of the isolates that were recognized as positive in the screening test on an inulin polyacrylamide gel. The obtained results of enzymatic activity are given in Table 1. *A. welwitschiae* strains FAW1, FAW6 and FAW9 have proven to be significant inulinase producers among *Aspergillus* section *Nigri*. *A. tubingensis* strains FAT30 and FAT34, and *A. niger* strains FAN1 and FAN3 had much lower inulinase activity.

The identified Serbian black aspergilli were tested for their potential to produce inulinases. For that purpose, we introduced a cheap and sensitive alternative inulinase producer screening test, which requires neither hazardous, growth restricting chemicals nor special equipment, and furthermore, yields conclusive results within 33 h. The described screening method represents an easy and fast way to detect potential fungal producers of inulinases. A large number of strains can be tested within a short period. The aim of developing this test was to decrease the time required to find and determine potential inulinase producers. Fermentation lasts only 33 h, which is enough time for fungi (*Aspergillus* spp.) to grow and to produce enzymes. A short fermentation production time is very important both to obtain a rapid response and to avoid sporulation, which is undesirable when dealing with a large number of fungal strains for several reasons: cross-contamination, safety of those performing the work, and possible false results resulting from changes in the enzyme production due to the advent of the next hyphae generation (*43*). Screening does not require protein purification; it uses the raw fermentation extract.

The idea for the second phase of the screening test originates from a pioneering enzymatic diffusion test in agarose gels (*44*). The diffusion test described here was done in PAA gel, which makes handling much easier and provides more precise and limited diffusion. Reaction products of inulin hydrolysis are reducing sugars which are retained in the PAA gel in places where inulin is hydrolyzed due to the precise and limited application of the enzyme to the wells. Detection of those small molecules would be difficult if the enzymes were applied to the surface of the agarose gel *via* filter paper as in other diffusion tests described (*44*, *45*). The aim of the developed diffusion screening test was to detect the activity of all inulinase enzymes (endoinulinase and exoinulinase) that act synergistically on inulin. Staining of the substrate on which the fungi grow only detects endoinulinase (*12*), which was not the aim of this research. Both inulinase enzymes are industrially important for obtaining inulooligosaccharides and fructooligosaccharides and they are usually both present in the fungal genome. The most commonly used spectrophotometric test for inulinase activity measures the hydrolysis product in this reaction, by which the activities of fungal fermentation extracts selected after screening were determined. Screening in inulin PAA gel reduced the number of required spectrophotometric analyses.

Among all tested species, isolates that produce inulinases as well as those that did not show the production of these enzymes were found. Most of *A. niger* and *A. welwitschiae*, and half of *A. tubingensis* isolates were inulinase producers. Among them, some strains of *A. welwitschiae* can be, by far, marked off as producers of higher amounts of inulinases (*i.e*. FAW1, FAW6 and FAW9). Since these species are known to be able to produce mycotoxins (*27*), determining their mycotoxin-producing potential, to ensure safe further usage of these isolates as producers of enzymes that will be used for food, was necessary. Therefore, *A. welwitschiae* isolates were subjected to further mycotoxin production analyses.

### Mycotoxin and inulinase production

The first step is certainly to determine whether mycotoxins are produced under conditions that are optimal for enzyme production. Solid-state fermentation (SSF) is a good choice for fungal enzyme production thanks to its advantages, such as low cost, high efficiency, reduced catabolic repression, easy maintenance, easy scale-up, *etc.* (*46*). Agro-industrial wastes and/or by-products are mainly composed of complex carbohydrates and crude proteins that can be useful as nutrients for microbial growth and as inducible substrates for enzyme production (*47*, *48*). For that purpose, we used natural substrate (triticale grains) for SSF, which previously proved good for inulinase production (*49*), to grow *A. welwitschiae* isolates and determine the presence of mycotoxins. The maximum inulinase production was determined on the 4th day of the production by five *A. welwitschiae* strains isolated from different sources and geographic regions, with different enzymatic activities, specific enzymatic activities and productivity (Table 2). It is known that different fungal sources (substrate composition and environmental conditions) have led to the production of different enzyme sets that actually allow them to grow in different habitats (*43*, *50*, *51*). Therefore, differences in inulinase amounts are expected here as well, since fungi originate from different habitats. FAW1 and FAW6 stood out, where FAW1 was the best (also shown in screening test) inulinase producer. Significant differences in the production of inulinase among isolates were verified by ANOVA test (F_4,10_=352.4, p<0.0001****, Table 2).

**Table 2:** Production of inulinase by *Aspergillus welwitschiae* isolates on triticale grains on the fourth day of SSF (maximum production)

Isolate	*γ*(protein)/ (mg/mL)	Inulinase activity/(U/mL)	Specific inulinase activity/(U/mg)	Productivity/ U/(mL·h)	*γ*(mycotoxin)/ (μg/mL)
FAW 1	(0.22±0.01)^a^	(10.4±0.8)^a^	(46.0±3.5)^a^	(0.11±0.01)^a^	0^a^
FAW 3	(0.33±0.01)^b^	(1.09±0.04)^b^	(3.4±0.1)^b^	(0.01±0.00)^b^	1.87^b^
FAW 4	(0.26±0.02)^bc^	(0.89±0.03)^bc^	(3.4±0.1)^bc^	(0.01±0.00)^bc^	2.62^c^
FAW 6	(0.33±0.02)^d^	(6.4±0.3)^d^	(18.9±1.0)^d^	(0.063±0.006)^d^	0^ad^
FAW 9	(0.309±0.001)^bce^	(1.45±0.09)^bce^	(4.6±0.3)^bce^	(0.013±0.006)^bce^	0.39^e^

Each data point represents the mean value of three independent assays±S.D. (standard deviation). Mean values followed by the same letter in superscript are not significantly different (Tukey’s; p<0.05)

FB2 production was confirmed in FAW2, FAW3, FAW4, FAW5 and FAW9 isolates using HPLC analysis of their fermentation fluids, while the production of FB1 was not detected in the assessed isolates (Table 3).

**Table 3:** Production of fumonisins (FB1 and FB2) and ochratoxin A (OTA), and mycotoxin biosynthetic gene amplification of *Aspergillus niger* and *A. welwitschiae* isolates examined in this study

Species	Isolate	*γ*(mycotoxin)/(µg/mL)				Fumonisin cluster genes						Ochratoxin cluster genes
FB1		FB2	OTA	*fum1*	*fum7*	*fum10*	*fum15*	*fum21 I*	*fum21 II*	*Ota 1,2,3,5*
*A. niger*	FAN 1	n.t.	n.t.		n.t.	+	+	+	+	+	+	-
*A. niger*	FAN 2	n.t.		n.t.	n.t.	+	+	+	+	+	+	-
*A. niger*	FAN 3	n.t.		n.t.	n.t.	+	+	+	+	+	+	-
*A. niger*	FAN 4	n.t.		n.t.	n.t.	+	+	+	+	+	+	-
*A. niger*	FAN 5	n.t.		n.t.	n.t.	+	+	+	+	+	+	-
*A. niger*	FAN 6	n.t.		n.t.	n.t.	+	+	+	+	+	+	-
*A. niger*	FAN 8	n.t.		n.t.	n.t.	+	+	+	+	+	+	-
*A. niger*	FAN 9	n.t.		n.t.	n.t.	+	+	+	+	+	+	-
*A. niger*	FAN 10	n.t.		n.t.	n.t.	+	+	+	+	+	+	-
*A. welwitschiae*	FAW 1	0		0	0	+	-	-	+	-	-	-
*A. welwitschiae*	FAW 2	0		2.85	0	+	+	+	+	-	+	-
*A. welwitschiae*	FAW 3	0		1.87	0	+	+	+	+	-	+	-
*A. welwitschiae*	FAW 4	0		2.62	0	+	+	+	+	-	+	-
*A. welwitschiae*	FAW 5	0		2.38	0	+	+	+	+	-	+	-
*A. welwitschiae*	FAW 6	0		0	0	+	-	-	+	-	-	-
*A. welwitschiae*	FAW 7	0		0	0	+	+	+	+	-	+	-
*A. welwitschiae*	FAW 9	0		0.39	0	+	+	+	+	-	+	-

n.t.=not tested

None of the tested *A. niger* and *A. welwitschiae* isolates contain any ochratoxin gene amplicons. HPLC analysis of fermentation fluids confirmed the results obtained by gene analysis and showed that the analyzed *A. welwitschiae* isolates do not produce ochratoxin under these growth conditions (Table 3).

All assessed *A. niger* and *A. welwitschiae* isolates were analyzed for the presence of six genes in *fum* cluster and four genes in *ota* cluster and the obtained results are presented in Table 3.

All *A. niger* isolates yielded amplicons for all six *fum* genes, while *A. welwitschiae* isolates yielded two amplicon patterns. The first *fum* amplification pattern of *A. welwitschiae*, present in two isolates (FAW1 and FAW6), consisted of *fum1* and *fum15* gene amplicons only, while the second, present in six isolates (FAW2, FAW3, FAW4, FAW5, FAW7 and FAW9) consisted of *fum1, fum7, fum10, fum15* and *fum21 II* gene amplicons. The *fum21 I* gene was not amplified in any of the assessed *A. welwitschiae* isolates (Table 3). Direct sequencing of the obtained *fum1* amplicons from *A. niger* and *A. welwitschiae* yielded nucleotide sequences 630-678 nt long, which were deposited in the NCBI GenBank under accession numbers given in Fig. 3 (*38*). For phylogenetic analyses based on partial *fum1* sequences, 19 *A. niger* and 18 *A. welwitschiae* from this publication and other previously described and characterized strains were selected. The unrooted *fum1* phylogenetic tree indicated clustering of *A. welwitschiae* strains into two highly supported separate clusters. One cluster encloses only fumonisin (FB)-non-producing strains, while the other cluster contained both, FB-producing and non-producing strains. However, no separate clusters of *A. niger* and *A. welwitschiae* were formed.

HPLC analysis of OTA showed that none of the assessed *A. welwitschiae* strains produced OTA, which was corroborated by the PCR analyses of* ota* gene cluster. Furthermore, our results confirmed a previously reported almost complete deletion of the *ota* cluster in OTA-non-producing *A. welwitschiae* (*29*). Although OTA-non-producers with incomplete deletion of *ota* cluster have been reported (*52*), complete deletion of *ota* cluster should imply the loss of ability to produce OTA under any conditions. The low percentage of OTA-producing strains among *A. welwitschiae* is not a surprise when compared to other studies where a minority of the strains have persistently shown to produce OTA. In most of the studies this percentage varies from 0 to 1% (*52*–*54*), while Susca *et al.* (*29*) reported 25%. Additionally, PCR analysis of *ota* cluster in *A. niger* suggests complete deletion, hence no producers of OTA among them either.

Unlike OTA gene analyses, which are fully consistent with Susca *et al.* (*29*), the analysis of FB revealed a slightly different situation. In our work, *A. welwitschiae* strains with incomplete *fum* gene cluster observed in the PCR pattern (compared to *A. niger* and f-1 pattern of *A. welwitschiae*) proved to be able to produce FB, although the incompletion was reflected as the presence of only partial amplification of the *fum 21* gene. In the work of Susca *et al.* (*29*), only *A. welwitschiae* strains with intact *fum* cluster (compared to *A. niger*) and f-1 *fum* gene amplification pattern were able to produce FB. The lack of amplification of *fum21 I* in FB-producing *A. welwitschiae* may not mean nonfunctional *fum 21*, since Sun *et al.* (*55*) showed that functional *fum 21* is essential for FB synthesis in *Fusarium proliferatum*. In Susca *et al.* (*29*), *fum* gene amplification patterns observed in the PCR assays were reported not to be consistent with the genomic sequences.

HPLC analyses of FB showed that out of eight assessed *A. welwitschiae* strains, five produced FB2, while three were non-producers of FB (*i.e.* FAW1, FAW6 and FAW7). *Fum* gene cluster analyses showed the presence of two patterns among *A. welwitschiae* strains. The presence of complete *fum* cluster genes, which is characteristic of FB-producers, was confirmed in six isolates. In two isolates (*i.e.* FAW1 and FAW6) some gene regions did not amplify and this is in agreement with Susca *et al.* (*29*), who found that multiple patterns of *fum* gene deletion are characteristic of FB-non-producing *A. welwitschiae*. In one strain (FAW7) the lack of FB production in the presence of complete *fum* cluster genes was detected. Two reasons can be given for such an outcome: (*i*) fumonisin production can vary under different conditions, hence it cannot be excluded that the strain could produce FB under different conditions, and (*ii*) production of FB could be affected by regulatory genes located outside the *fum* cluster, as it is the case for *Fusarium* sp. (*56*). Also, we analyzed the obtained *fum1* gene sequences, which is considered as the key gene in the FB biosynthetic pathway, that encode polyketide synthase (*57*). The results obtained in this work are consistent with the previously published results of phylogenetic analysis of full-length *fum1* and *fum15* nucleotide sequences separately and concatenated (*28*). The partial *fum1* nucleotide sequence phylogeny inferred in this work showed separation of *A. welwitschiae* strains into two well supported clades, one of which consisted solely of FB-non-producing, while the other consisted of both FB-producing and -non-producing strains.

Strain FAW7 was initially selected as a good inulinase producer and it did not coproduce FB under selected conditions, but its mycotoxin potential represents a substantial risk for its use as a producer of enzymes for food. It is encouraging that among the selected inulinase producers, two (FAW1 and FAW6) are non-toxigenic (both OTA and FB) and they have a potential for further evaluation and possible use for inulinase production.

## CONCLUSIONS

It is important to be certain that the producers of enzymes that are used in food industry do not produce mycotoxins during enzyme production, or even better, are not capable of producing mycotoxins at all. The latter can be achieved by using nontoxigenic species or strains that lack fully or partly the genetic repertoire for biosynthetic pathways of mycotoxin production. Only by combining all of the presented results (possibility of fungi to secrete mycotoxins and enzymatic activity) can it be concluded whether a strain is a potentially good enzyme producer. We selected two strains of *Aspergillus welwitschiae* (FAW1 and FAW6) for further development for enzyme production as the best inulinase producers and mycotoxin non-producers, with the absence of the whole *ota* cluster and essential components of the *fum* cluster as additional safety.
